# Wearable Feet Pressure Sensor for Human Gait and Falling Diagnosis

**DOI:** 10.3390/s21155240

**Published:** 2021-08-03

**Authors:** Vytautas Bucinskas, Andrius Dzedzickis, Juste Rozene, Jurga Subaciute-Zemaitiene, Igoris Satkauskas, Valentinas Uvarovas, Rokas Bobina, Inga Morkvenaite-Vilkonciene

**Affiliations:** 1 Department of Mechatronics, Robotics, and Digital Manufacturing, Faculty of Mechanics, Vilnius Gediminas Technical University, LT-03224 Vilnius, Lithuania; vytautas.bucinskas@vilniustech.lt (V.B.); juste.rozene@vilniustech.lt (J.R.); jurga.subaciute-zemaitiene@vilniustech.lt (J.S.-Z.); 2 Clinic of Rheumatology, Orthopaedics Traumatology and Reconstructive Surgery, Faculty of Medicine, Vilnius University, LT-03101 Vilnius, Lithuania; igoris.satkauskas@mf.vu.lt (I.S.); valentinas.uvarovas@mf.vu.lt (V.U.); rokas.bobina@mf.stud.vu.lt (R.B.); 3 Centre of Orthopaedics and Traumatology, Republican Vilnius University Hospital, Šiltnamių Str. 29, LT-04130 Vilnius, Lithuania

**Keywords:** feet pressure sensor, human gait, falling diagnosis

## Abstract

Human falls pose a serious threat to the person’s health, especially for the elderly and disease-impacted people. Early detection of involuntary human gait change can indicate a forthcoming fall. Therefore, human body fall warning can help avoid falls and their caused injuries for the skeleton and joints. A simple and easy-to-use fall detection system based on gait analysis can be very helpful, especially if sensors of this system are implemented inside the shoes without causing a sensible discomfort for the user. We created a methodology for the fall prediction using three specially designed Velostat^®^-based wearable feet sensors installed in the shoe lining. Measured pressure distribution of the feet allows the analysis of the gait by evaluating the main parameters: stepping rhythm, size of the step, weight distribution between heel and foot, and timing of the gait phases. The proposed method was evaluated by recording normal gait and simulated abnormal gait of subjects. The obtained results show the efficiency of the proposed method: the accuracy of abnormal gait detection reached up to 94%. In this way, it becomes possible to predict the fall in the early stage or avoid gait discoordination and warn the subject or helping companion person.

## 1. Introduction

According to the World Health Organisation (WHO), falls are the second leading cause of accidental deaths globally, with 37.3 million deaths. A total of 30% of people over 65 fall at least once a year, and this number increases with age [1,2]. Factors leading to the onset of falls in older age are: culture and gender, determinants related to health and social services, physical and social environments, as well as personal factors (attitudes, fear of falling, coping with falls, ethnicity, and race), economic and behavioural (physical activity, healthy eating, use of medicines, risk-taking behaviours) determinants [1,2]. Moreover, the analysis of the causes of falls in the elderly revealed an association between postural stability, control and reflexes, and physiological and neuromuscular changes with decreased muscle strength and amplitude of lower limb joint movements [3], displacement of the centre of gravity [4], changes of the vision [5], head injuries, bone fractures, joint distortions and dislocations, and soft-tissue bruises, contusions, and lacerations [6,7,8]. All the factors become a cause of dangerous and consequent falls. Therefore, detecting changes in human gait by simple, cheap, and effective methods is a fundamental issue. The main parameters analysed to determine ways to prevent or delay falls are becoming essential: balance, posture, gait, locomotion, gait variability, gait disorders, gait disturbance, elderly, ageing, falls, and vision [5].

Various systems were used for human gait analysis: 3-D-acceleration sensors with data logger and a computer program for processing the acceleration signals and calculating the gait parameters [2]; Vicon 3D motion capture system, with 10 digital cameras and the reflective markers for measuring the movements of the segments of lower extremities, and two sequentially staggered AMTI force platforms measuring ground reaction forces [8]; insole-force sensors used for gait analysis and wireless plantar force measurements in hopping, walking, and running [9]; Vicon 370 system with strobe cameras, force plates, reflective markers, and Vicon Clinical Manager software used for gait analysis, and the handheld dynamometer for the muscle strength [10]. These methods are effective and accurate; however, they are too expensive and complicated to use in everyday activities. The other solution could be implementing flexible tactile sensors based on polymeric composite materials [11,12,13] exhibiting piezoresistive properties. Flexible piezoresistive sensors are used for the evaluation of pressure or applied normal force in non-stiff objects [14,15,16,17], in some robotics issues [18,19,20,21], and as wearable sensors [22]. The polymeric materials in such sensors must be biocompatible, mechanically and chemically stable, and reliable. To use these materials in sensors for everyday life, they also should be relatively inexpensive and freely available on the market. The auspicious polymeric composite material for such application is Velostat^®^, also known as Linqstat [23,24]. Velostat^®^ consists of carbon-impregnated polyethylene, which makes it an electrically conductive piezoresistive material, applied in the design of flexible sensors [24,25,26,27]. The resistive sensors are of low manufacturing cost compared to other types of sensors. Required interfacing circuits and data acquisition processes are simple and often preferred in various applications. A more detailed description of Velostat^®^-based sensors, their mechanical and electrical properties, working principle, and applications can be found in our previous paper [27]. We showed that it is possible to use such sensors as lifelong and reliable devices where absolute accuracy is not a crucial parameter. The determined mechanical and electrical properties of the material and the load-unload deflection behaviour revealed sensor reliability and signal drift issues.

The aim of this article is to provide methodology and equipment for human gait and falling diagnosis without limiting the human’s everyday activities. To predict the fall in the early stage or to avoid gait discoordination and warn the subject or helping companion person, we proposed three specially designed Velostat^®^-based wearable feet sensors installed in the shoe lining solution.

## 2. Formulation of the Research

**Figure 1:**
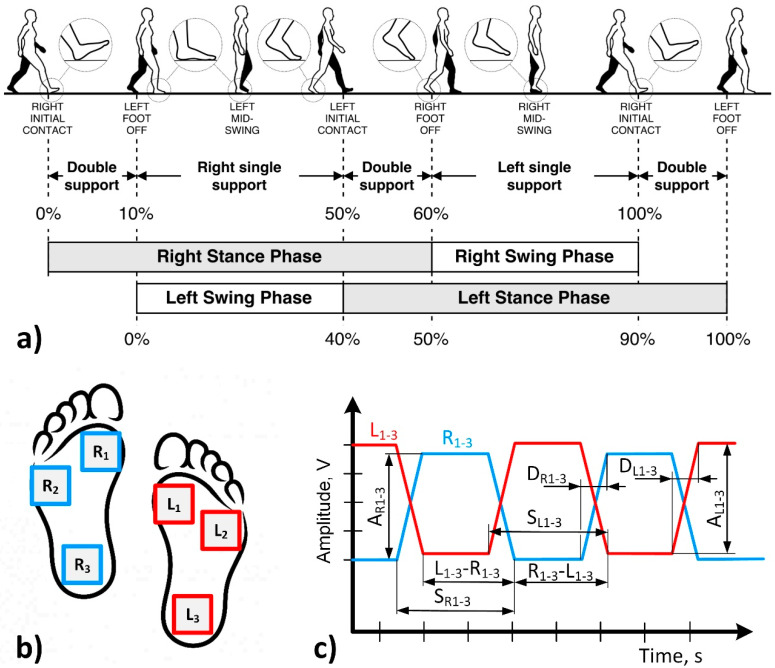
Scheme of human gait analysis: (**a**) human gait phases [28,29]; (**b**) sensors placement; (**c**) extraction of main parameters from sensors signals. L_1–3_; signals from left foot sensors, R_1–3_; signals from right foot sensors, A_R1–3_ and A_L1–3_; parameters representing weight distribution on the right and left feet, S_R1–3_ and S_L1–3_; duration of left and right stance phases, D_R1–3_ and D_L1–3_; stepping abruptness, L_1–3_–R_1–3_ and R_1–3_–L_1–3_; stepping unevenness.

Typically, a human’s gait consists of four main phases (Figure 1a): right stand, right swing, left stand, and left swing [28,29]. The standing phase includes cases when the human body is supported by two legs, and then the human stands on one leg. This phase takes about 60% of one step duration: 20% for both legs support and 40% for standing on one leg. The swing phase takes the remaining 40% of the step duration and defines the weight distribution process from one leg to another. Non-rhythmic variation in phase distribution and durations and unequal load distribution on the feet notify gait discoordination and fall possibility.

There are many methods and a lot of available equipment to detect gait discoordination in specialised laboratory conditions. Still, those methods only suit short-term monitoring and investigations since they are not applicable in causal activities. To overcome those limitations, it is necessary to develop an automated gait analysis and warning system that will not disturb human mobility and will be comfortable and straightforward to use in everyday life. The basis of such a system is the gait analysis sensors and their data processing methods.

To detect gait phases, we propose the use of six force (Figure 1b) sensors installed in the shoe linings (three sensors for each foot). Such a solution allows simultaneous detection of all gait phases and load distributions on the feet, which is essential in evaluating gait discoordination. Such sensors will form pulse sequences whose periods and amplitudes correspond to the duration of gate phase and load distribution on feet, respectively (Figure 1c). Comparing signals from both feet makes it possible to detect non-rhythmic or unequal steps leading to gait discoordination and possible falls. Moreover, such a method can be applied to detect congenital gait defects by analysing feet load distribution.

We developed specialised low cost Velostat^®^-based wearable feet sensors suitable to mount under the lower surface of the simple shoe lining for feet load measurements. Velostat^®^ as a sensitive sensor element was selected due to a few reasons: firstly, due to its piezoresistive nature, resistive sensors are well known, and they do not require specialised readout hardware; secondly, it allows the manufacture of thick film type sensors with custom-sized surface area; implementation of such sensors is simple and can be performed by an unqualified consumer. Moreover, the proposed method is based on detecting relative changes in sensors response signals. Analysing sensors output signals, we do not compare absolute values but differences between the states when a foot is on the ground and when it is raised. Therefore, the proposed method is insensitive to installation misalignments of a few millimetres and does not require calibration to relate resistance variation with an absolute value of pressure or load.

**Figure 2:**
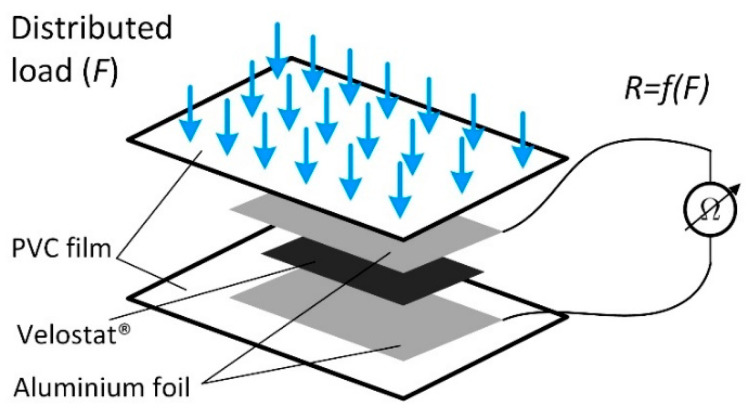
Structure of the developed wearable foot sensor.

The developed sensor (Figure 2) is a sandwich-type structure in which a piece of Velostat^®^ 20 × 20 mm is placed between electrodes made from 100 µm thickness aluminium foil. The position of the Velostat^®^ and the electrodes are fixed using about 150 µm thick adhesive PVC film, which at the same time acts as the sensor housing and protects it from the direct electric circuit and external influences, such as variation of humidity.

The remaining part of our paper is intended to prove the functionality of the proposed human gait and falling diagnosis method by evaluating various cases of human gait discoordination.

## 3. Experiments

The effectiveness of the developed sensor and the proposed method were researched by performing physical experiments in scientific laboratories of the Mechatronics, robotics, and digital manufacturing department at Vilnius Gediminas technical university according to recommendations provided by colleagues from the Republican Vilnius University Hospital.

### 3.1. Experimental Setup

**Figure 3:**
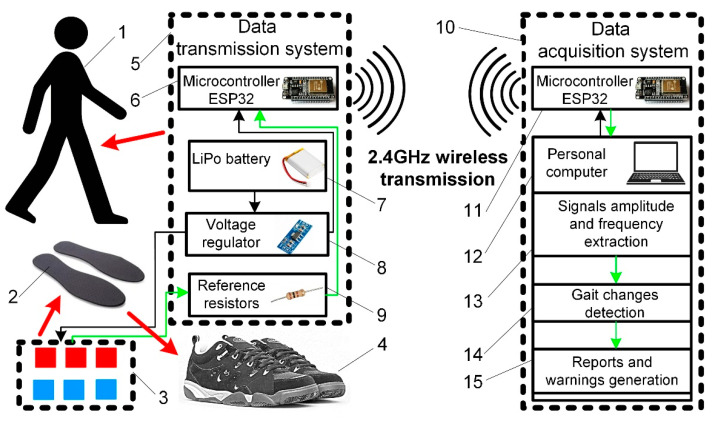
Experimental setup: 1; potential patient, 2; shoe linings, 3; feet sensors, 4; shoes, 5; data transition system, 6; microcontroller, 7; battery, 8; voltage regulator, 9; reference resistors, 10; data acquisition system, 11; microcontroller, 12; personal computer, 13; parameters extraction algorithm, 14; gait changes detection algorithm, 15; reports and warnings generation function. Black lines represent power supply lines, and green represent data flow.

To perform experiments under conditions as close to real ones as possible, we developed a particular experimental setup (Figure 3).

The developed gait analysis system consists of sensors and two main modules: the data transmission module, which acts as a wireless transmitter, and the data acquisition module acting as receiver and data logger.

A total of six piezoelectric pressure sensors of the original design, made of Velostat^®^ PVC film that protects them from external influences, were used in the experiment. The sensors were attached to the lower surface of the universal shoe lining (Figure 1b) using a general-purpose adhesive film. Such installation ensures the potential patient’s minimal discomfort level since there are no direct contacts between the sensor surface and the human body in the system. A minor inconvenience is only the thin, flexible wires from the sensors, but they are designed not to restrict human movement.

All sensors were connected to the data transmission system, consisting of a microcontroller mounted in a unique 3D printed PLA (polylactic acid) plastic housing/box made to be attached to clothing or placed in a pocket. The data transmission module consists of an ESP32 microcontroller with an integrated wireless interface, a sensor connection circuit operating on a voltage divider scheme with reference resistors of 200 Ω, and a 7.4 V 1300 mAh lithium polymer battery providing system power. This module is used for sensor data acquisition and transmission via a wireless 2.4 GHz WIFI interface. Technical solutions based on wireless data transmission reduces the amount of equipment a person needs to carry and ensures free movement in the relatively large area around the data acquisition system. For example, the receiver can be placed anywhere in the house while the patient moves between the separate rooms. Moreover, the transmission system is compatible with all WIFI devices and can be adjusted to send data to tablets or smartphones.

The data acquisition module consists of an ESP8266 microcontroller connected to a personal computer via a USB interface. This module acts as a receiver and is responsible for receiving the data sent by the master controller and transmitting it to the personal computer. The obtained data is stored in the MS Excel database, where it is further processed by analysing the changes in the parameters describing the gait.

### 3.2. Methodology

Our experiments were focused on proving the functionality of the developed sensors and the proposed method. Therefore, we focused on the possibility of detecting gait changes by analysing our selected parameters. For this purpose, we performed experiments simulating various gait changes. We recorded the gait sequence consisting of more than four full steps of one person under six different cases: turnaround, scrolling, climbing upstairs, climbing downstairs, climbing upstairs one by one, walking with one straight leg. Each experiment was repeated three times to compile a dataset consisting of 18 graphs for further analysis (see supplementary data). There was a 5 min break before each experiment and its repetition to avoid the impact of fatigue.

The acquired data were analysed in a semi-manual mode using standard features available in MS Exel. From sensors signals, we extracted all parameters shown in Figure 1c. Firstly, we analysed load distribution over the feet during all six simulated cases. The obtained results proved our initial hypothesis. Therefore, for further validation, we selected the two most often examples of gait discoordination: scrolling and walking with one straight leg. Secondly, we evaluated the duration of the left and right stance phases. Thirdly, we defined variation of stepping abruptness since this parameter provides reliable information about gait robustness. Finally, we extracted parameters representing stepping unevenness as a shift in time between the left and right stance phase.

All obtained numerical values were averaged and statistically processed according to the below-provided methodology.

### 3.3. Calculations

The confidence of the obtained results was evaluated by statistical parameters using correlation-regression analysis. Arithmetic averages, their standard deviations, and confidence intervals at 0.95 probability level were calculated according to the methodology provided in [30].

In correlation-regression analysis, absolute measurement error was defined as the difference between the measured result *m_v_* and the real measured parameter value *r_v_* [30]:

(1) $$∆m_{v}=m_{v}-r_{v}$$

The measured parameter m_*v*_ has a prescribed probability of representing the real measured value if the same measurement is repeated *n* times. The arithmetic mean from several measurements $\bar{m_{v}}$ can be calculated as:

(2) $$\bar{m_{v}}=\frac{1}{n}\sum_{i=1}^{n}m_{vi}=\frac{m_{v1}+m_{v2}+\ldots m_{vn}}{n}$$

Absolute measurement error consists of systematic, random, and random errors of deduction. Using correlation-regression analysis, only random errors are estimated in statistical data evaluations. In our case, we evaluated only random errors, as if the methodology and measurement devices are far more accurate than expected error, systematic and accidental deduction errors are not significant and therefore can be not considered [30]. To evaluate random error, it is necessary to calculate the experimental standard deviation *σ* of each measurement:

(3) $$\sigma =\sqrt{\frac{\sum_{i=1}^{n}{\left(m_{v}-\bar{m_{v}}\right)}^{2}}{n-1}}.$$

Experimental standard mean deviation *S_md_* is calculated by:

(4) $$S_{md}=\frac{\sigma }{\sqrt{n}}$$

The random error of the measured parameter is calculated by:

(5) $$∆m_{v,n,P}=S_{c}\cdot S_{md}$$

where: *S_c_* = the value of the student criterion selected according to the number of experiment variables (*n* − 1) and the probability level (α = 0.95). The final result of the measured (*n* times) value *m_v_* is expressed as the sum of the arithmetic mean *m_v_* and the random error Δ*_mv_*_,*n*,*P*_:

(6) $$\bar{m_{v}}\pm ∆m_{v,n,P}$$

## 4. Results

### 4.1. Analysis of Feet Load Distribution

**Figure 4:**
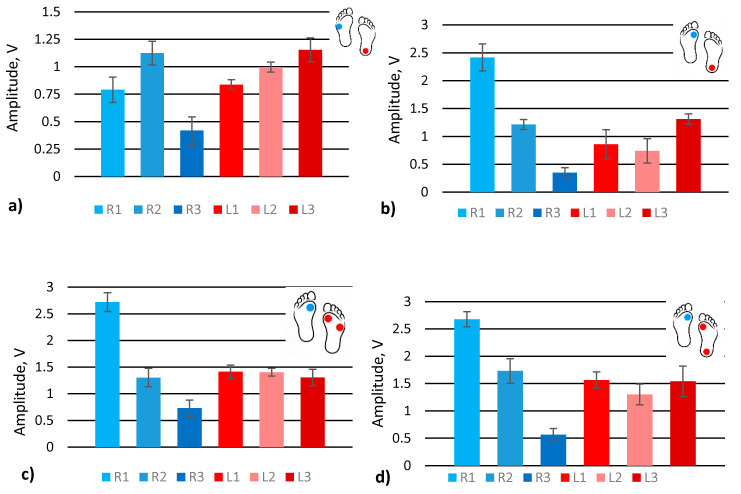

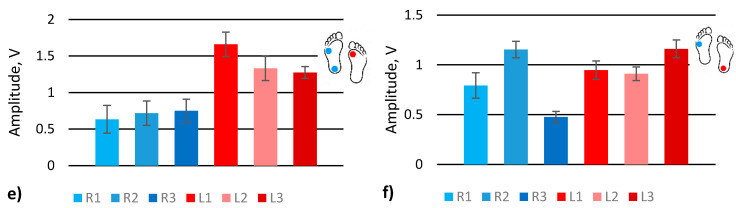
Analysis of signal amplitudes obtained from simulating various gates: (**a**) turnaround; (**b**) scrolling; (**c**) upstairs; (**d**) downstairs; (**e**) upstairs one by one; (**f**) walk with left straight leg.

Feet load distribution (Figure 1c A_R1–3_ and A_L1–3_) at different gaits, turnaround (Figure S1), upstairs (Figure S2), downstairs (Figure S3), scrolling (Figure S4), upstairs one by one (Figure S5), and walking with one straight leg (Figure S6), were evaluated by extracting signal amplitude values (Figure 4) from the raw data provided in the supplementary data. The graphs show the averaged amplitude values of each sensor that corresponds to the load generated by the feet. Dots shown on the feet symbol represent the position of the highest measured pressure on the feet.

The graph shows that the turnaround was performed by turning on the left foot heel and supporting the body with the right foot (Figure 4a). Sensors R2 and L3 are of the highest activity and clearly show the nature of the actual gait. In addition, the left foot sensors have higher signal amplitude than right foot sensors since the turnaround was performed standing on the left foot.

In the case of scrolling, sensor R1 shows the highest activity, as it should be for this type of gait (Figure 4b). The upstairs and downstairs gaits look similar. The highest activity is at the R1 sensor since the major part of the body load is distributed on the front of the foot (Figure 4c,d). For the left foot, in this case, all the sensors show similar activity. Nevertheless, it can be noted that in the case of scrolling, the main load of the left leg is placed on the heel while climbing upstairs, and the downstairs left foot load is distributed almost equally among all sensors.

The gait of upstairs one by one was performed by stepping one leg and then stepping with the other leg to the same step of stairs (Figure 4e). The left foot was the first, on which a human stands while moving the other one. The sensors L1, L2, and L3 show that a major part of the load was placed on the left foot, especially the front part, while the right foot was loaded almost equally. However, there is an exceptional tendency to load the heel of the right foot a little more than the front part of the same foot. Walking with the left straight leg shows the heel’s activity, while on the right foot, the highest activity is on the front sensor R2 (Figure 4f), similar to in the turnaround case.

In conclusion, feet load distribution and its variation due to natural obstacles or gait changes can be distinguished by analysing the amplitudes of the signals from pressure sensors placed in the shoe lining. If human gait changes, for example, from up or downstairs to upstairs one by one, the effect will be distinguishable from the normal gait. However, the analysis of load distribution is insufficient to determine the reason for the change in gait unambiguously. Other gait cycle parameters must also be evaluated to have a clearer picture.

### 4.2. Analysis of Gait Phases Duration

**Figure 5:**
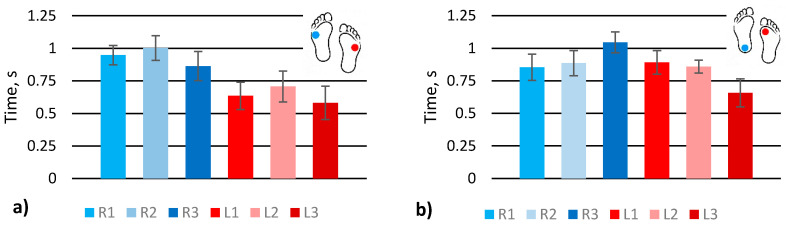
Analysis of stance phase duration at various gaits: (**a**) scrolling; (**b**) walk with one straight leg.

Analysis of signal amplitudes proves the functionality of our sensor and its sufficient sensitivity and response time. For further analysis, we selected two gaits (scrolling and walking with one straight leg) that often occur in the case of older or disease affected adults (Figure 5). The values of SR_1–3_ and SL_1–3_ (Figure 1c) were extracted from the supplementary data. In the scrolling case, the sensors’ behaviour looks similar, comparing one foot’s sensors with another, but the stance time differs: the right foot presses the sensors longer than the left leg. The gait with one straight leg shows that the sensor at the heel R3 is active longer since the straight leg does not allow the pressuring of all the sensors in the same manner. The L1 sensor at the foot front is activated longer when a human stands on the other leg to balance the walk.

**Figure 6:**
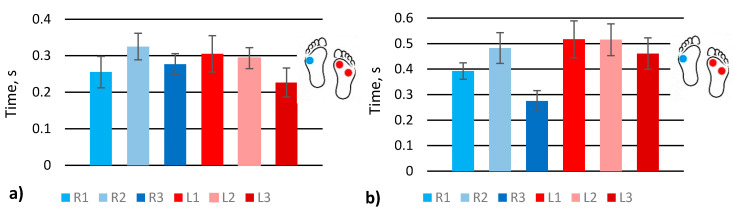
Analysis of stepping abruptness at various gaits: (**a**) scrolling; (**b**) walk with one straight leg.

The stepping abruptness indicates gait certainty, shows how fast a human places their foot, from initial contact to full loading, since it is the time between a pressed and unpressed sensor (Figure 1c, D_R1–3_, D_L1–3_). The longest time, as expected, is shown by front sensors in both gaits (Figure 6) because typically, in normal gait, initial contact with the ground is done with the front part of the foot. Nevertheless, simulating abnormal gait, it is seen that initial contact was detected by sensors R2 and L2 instead of sensors R1 and L1.

**Figure 7:**
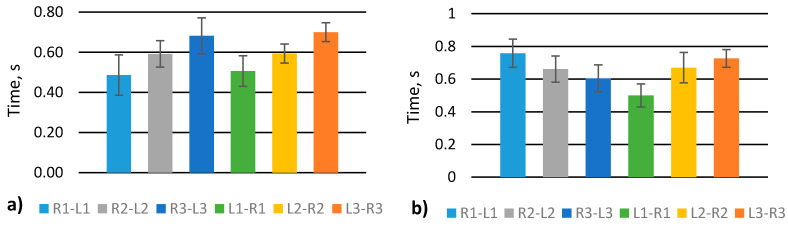
Analysis of stepping unevenness of various gaits: (**a**) scrolling; (**b**) walk with one straight leg.

Stepping unevenness was evaluated by extracting L_1–3_–R_1–3_ and R_1–3_–L_1–3_ parameters (Figure 1c). A scrolling gait shows that the signal increases from 1st to 3rd sensors, i.e., from front to heel, and the signal is similar for the same sensors. For example, R1–L1 is practically the same as L1–R1 (Figure 7a). On the contrary, one straight leg walk shows that the signal decreases from 1st to 3rd sensors for R–L measurement and increase from 1st to 3rd for L–R measurement. It means that scrolling almost does not impact stepping rhythm contrarily to walking with one straight leg.

The gait parameters detected by various methods and different types of sensors are provided in Table 1. Some of the methods implement IMUs [28,29,31], accelerometers [2,32], or reflective markers mounted on the body. In the case of IMUs or accelerometers, a huge processing power is needed to define essential gait parameters in real-time. On the other hand, the measurement result will depend on the mounting place; therefore sensor placement requires some measurement skills. In the case of reflective markers, it is necessary to use stationary cameras to detect the positions of the markers [8,10], which makes this method impossible to use in everyday life. In the case of the proposed sensor skill, computing power and limited space restrictions are not applicable. Sensors can be placed in any type of shoe using a universal shoe lining; also, it is not sensitive to sensor placing inaccuracy and can be replaced by the user. It is relatively cheap and does not requires additional mounting elements, such as a belt or elastic band. Moreover, the proposed solution uses simpler data acquisition and analysis than IMUs or vision-based systems. Furthermore, it is better suited for long-term gait monitoring in everyday activity without human comfort and mobility limitations.

## 5. Conclusions

The pressure sensor, designed and developed in our lab [27], was applied for the human gait diagnosis by placing three sensors in each shoe lining and measuring the response. The pressure distribution of the feet allows the analyses of the human gait by evaluating the main parameters: stepping rhythm, size of the step, weight distribution between heel and foot, and timing of the gait phases. The gait mode can be detected by analysing sensor signals in the time domain and extracting main parameters, such as pulse amplitude, pulse width, steepness of the pulse front edge, and shift between pulses. Analysing weight distribution in feet, the most significant difference observed in amplitude between signals from one-foot sensors was ~2 volts. The largest difference detected comparing the signals from both legs was ~1 volt. Respectively, analysing step phase parameters, the average difference between one leg signals was ~0.15 s, the difference between both legs sensors reached ~0.25 s. Analysis of stepping unevenness in the case of walking with one straight leg showed the step phase difference of ~0.3 s while stepping from right leg to left and vice-versa. The obtained results prove that we selected suitable parameters for data analysis, and the proposed system is applicable for the automated detection of gait variation.

Moreover, the proposed method and equipment setup is universal and adjustable according to the individual case. Even analysis of solely signal amplitudes shows some gait variations. If human gait will change, for example, from up or downstairs to upstairs one by one, the effect will be distinguishable from a normal gait. Furthermore, our method also suits clinical applications and can define gait changes during the rehabilitation process after trauma or invasive surgery.

This type of sensor can be implemented directly in the shoe insoles for the control of therapies, curing after surgery, or control of human gait for senile or disease damaged patients. The analysis of sensor signals can be automatised and performed directly by a mobile phone application, which can deliver results to a specialised server for observation or instant help. Shoe insoles with sensors in mass production will have a marginal increase of price, data transmitting and analysis devices and software brings affordable prices even for old or handicapped persons as well as social services.

This research proves the functionality of the proposed method and developed sensors. Nevertheless, future development should include the creation of automated data extraction and analysis algorithms, implementation of the warning system, and method validation with a sufficient number of tests, including peoples of different ages and health conditions.

## Figures and Tables

**Table 1:** The methods used for the gait disturbances researches.

Sensor Type and Placing	Method	Gait Parameters	Ref.
Accelerometers in belt on the waist.	Time measure, vectorial calculations, FFT, autocorrelation, wavelet analysis, and statistical regressions.	Global kinetic behaviour of the gait.	[2]
Reflective markers on the body and cameras. Two sequentially staggered AMTI force platforms.	The 3D kinematics calculated by the Euler angle theorem and inverse dynamics. Statistical analyses.	Gait speed, stride length and width, cadence, stance, hip extension, hip flexion, knee extension, knee flexion, ankle dorsiflexion, ankle plantarflexion.	[8]
Reflective markers, cameras, and force plates placed in the middle of a walkway. Handheld dynamometer.	The 3D kinematics calculated by commercial software. Statistical analyses.	Spatiotemporal, kinematic, and kinetic variables of gait, strength of hip flexor, adductor, and abductor’s muscles.	[10]
IMUs placed on the upper surface of shoe or feet	The MLA and Kalman filtering. IC/FO detection algorithm.	A rich set of standard spatio-temporal gait metrics.	[28]
Seven different IMU sensors, OptoGait measurement system.	The MLA and Kalman filtering, double integration of acceleration measurements.	Stride length, stance and swing times, and walking speed.	[29]
Custom assembled IMUs on feet, shank, and thigh.	The MLA with data fusion.	Stride length, stride speed, stride frequency, walking cycle, stance time, swing time, clearance, and knee ROM.	[31]
IMU sensor on bare foot.	The MLA with sensor fusion and Kalman filter. Double integration of acceleration data.	Stride distance, speed, length, and period. The ratio and phases between stance and swing.	[33]
Insole equipped with pressure sensors and a triaxial accelerometer.	Gait data recorded as time series signals.	Heel strike, foot flat, mid-stance, heel off, toe-off, mid-swing, and late swing.	[32]
Insole-Force Sensors.	Shapiro–Wilk-Test, Mann–Whitney-U-Test, Statistical analyses.	Loading of the lower limbs.	[9]
Six original flexible piezoresistive pressure sensors attached to the lower surface of the universal shoe lining.	Signal analysis in the time domain. Statistical analysis.	Feet load distribution, stance phase duration, stepping abruptness, and stepping unevenness.	This article

IMU—The inertial measurement unit; MLA—machine learning algorithm; IC/FO—initial-contact/foot-off.

## Data Availability

Not applicable.

## Supplementary Materials

The following are available online at https://www.mdpi.com/article/10.3390/s21155240/s1, Figure S1: Turnaround gate phase, first, second and third try (a–c), Figure S2: Upstairs gate phase, first, second and third try (a–c), Figure S3: Downstairs gate phase, first, second and third try (a–c), Figure S4: Scrolling gate phase, first, second and third try (a–c), Figure S5: Upstairs one by one gate phase, first, second and third try (a–c), Figure S6: Walk with one straight leg gate phase, first, second and third try.
