# Supplementary material for: Wearable Feet Pressure Sensor for Human Gait and Falling Diagnosis
Source: Sensors (Basel). 2021 Aug 3;21(15):5240. doi: 10.3390/s21155240 (PMC8347941; doi:10.3390/s21155240)
Supplement: Supplementary file 1 [file sensors-21-05240-s001.zip › sensors-1300962-supplementary.pdf]

sensors

Supplement

Wearable feet pressure sensor for human gait and falling diagnosis

Vytautas Bucinskas, Andrius Dzedzickis, Juste Rozene, Jurga Subaciute-Zemaitiene, Igoris Satkauskas, Valentinas Uvarovas, Rokas Bobina and Inga Morkvenaite-Vilkonciene

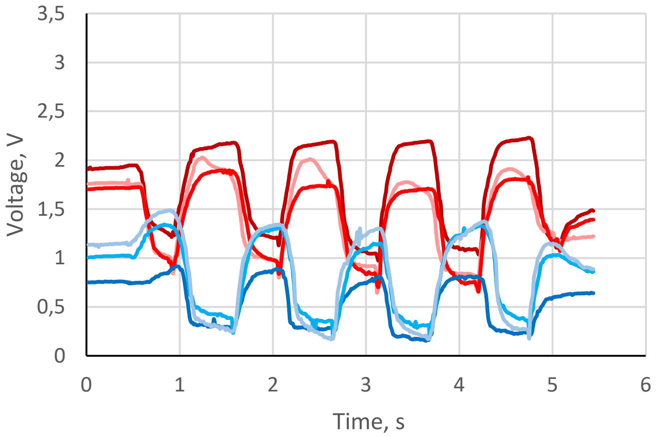

a)

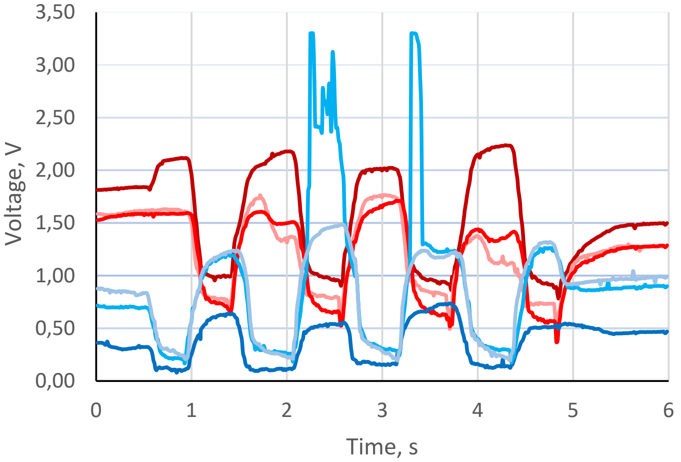

b)

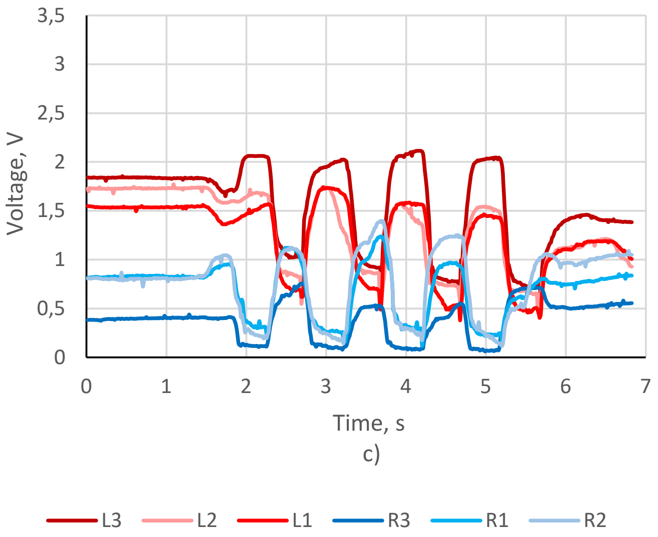

Figure S1: Turnaround gate phase, first, second and third try (a, b, c)

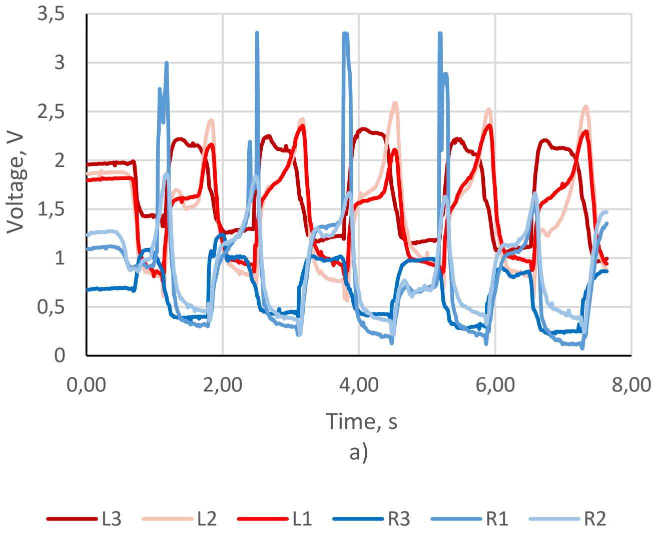

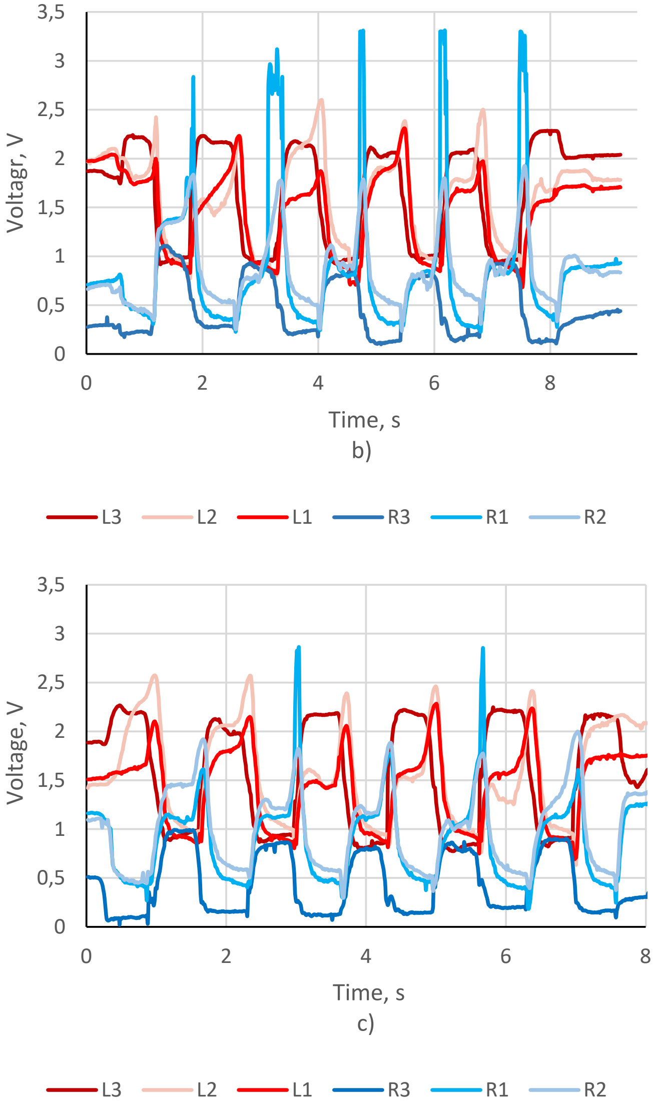

Figure S2: Upstairs gate phase, first, second and third try (a,b,c)

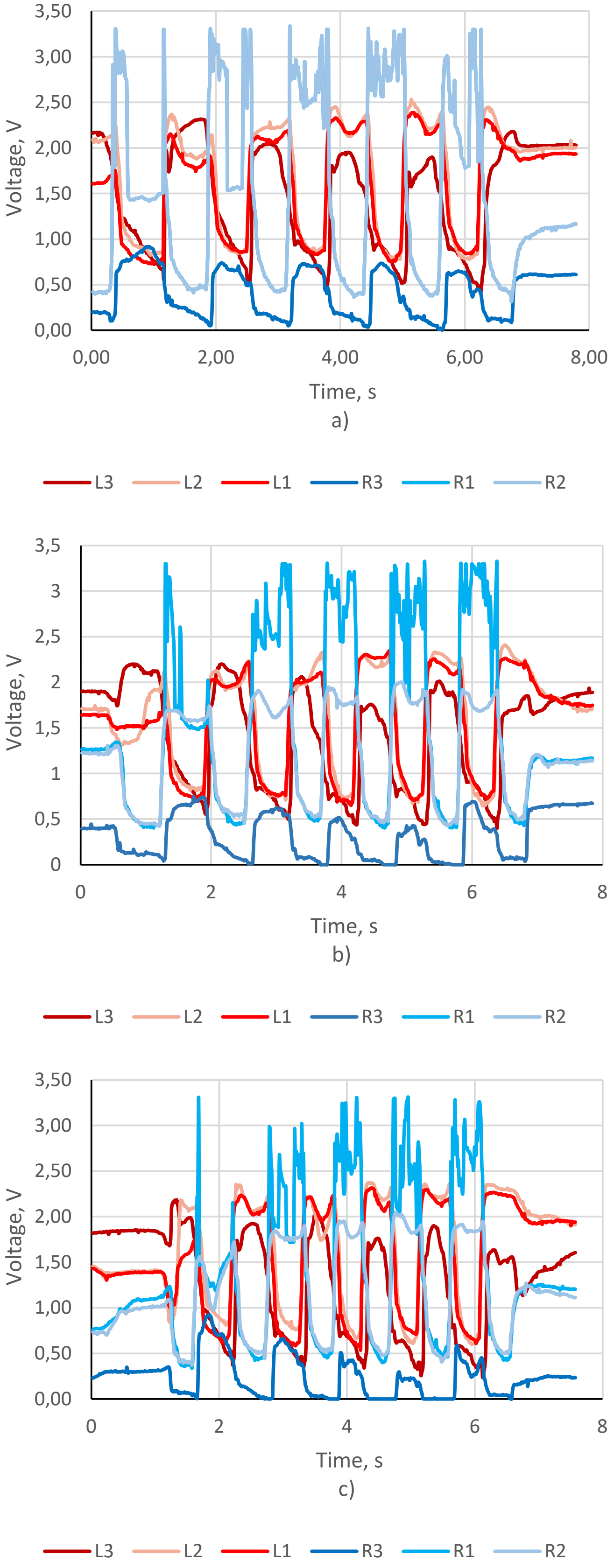

3,50
3,00
2,50
2,00
1,50
1,00
0,50
0,00
Voltage, V
0,00
2,00
4,00
6,00
8,00
Time, s
a)
L3
L2
L1
R3
R1
R2
3,5
3
2,5
2
1,5
1
0,5
0
Voltage, V
0
2
4
6
8
Time, s
b)
L3
L2
L1
R3
R1
R2
3,50
3,00
2,50
2,00
1,50
1,00
0,50
0,00
Voltage, V
0
2
4
6
8
Time, s
c)
L3
L2
L1
R3
R1
R2

Figure S3: Downstairs gate phase, first, second and third try (a,b,c)

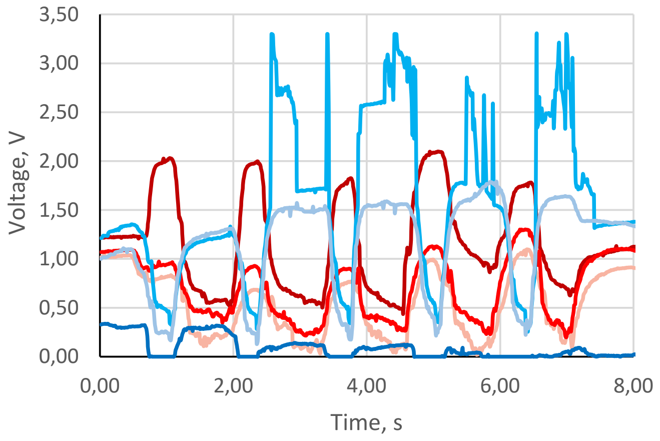

a)

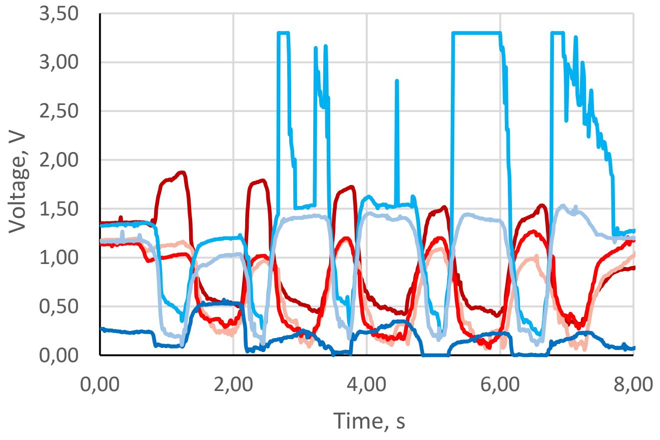

b)

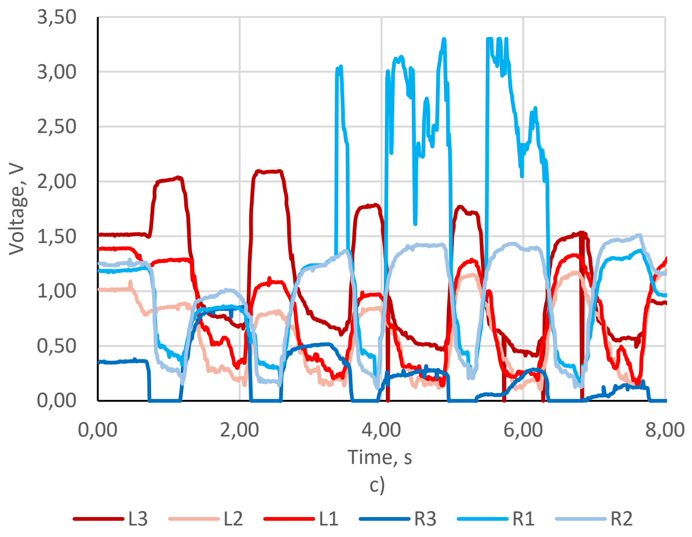

Figure S4: Scrolling gate phase, first, second and third try (a,b,c)

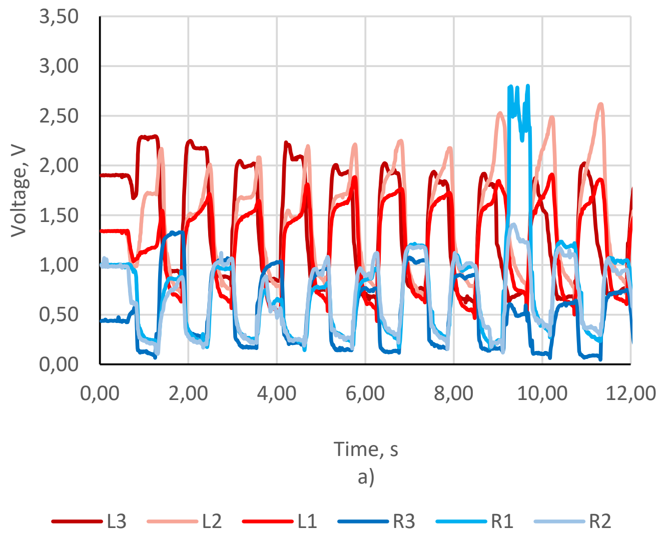

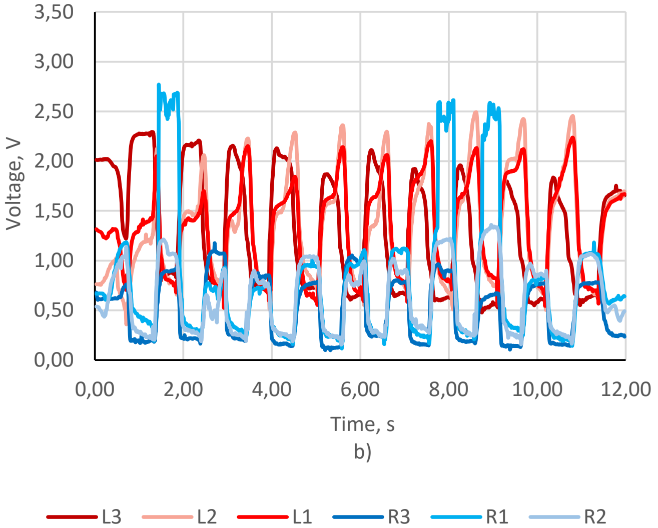

b)

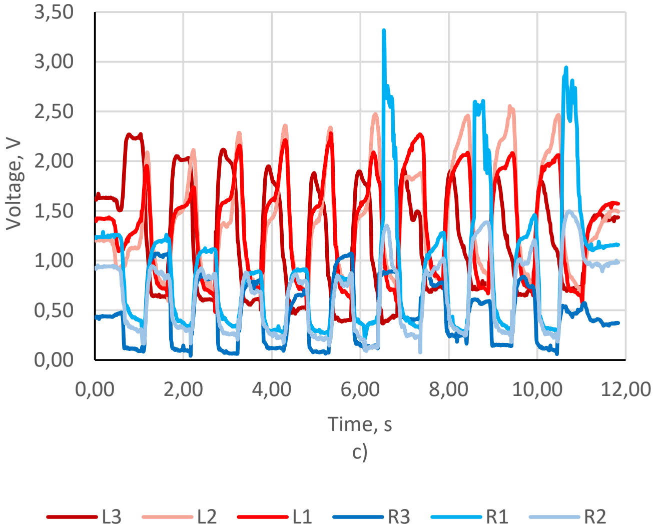

c)

Figure S5: Upstairs one by one gate phase, first, second and third try (a,b,c)

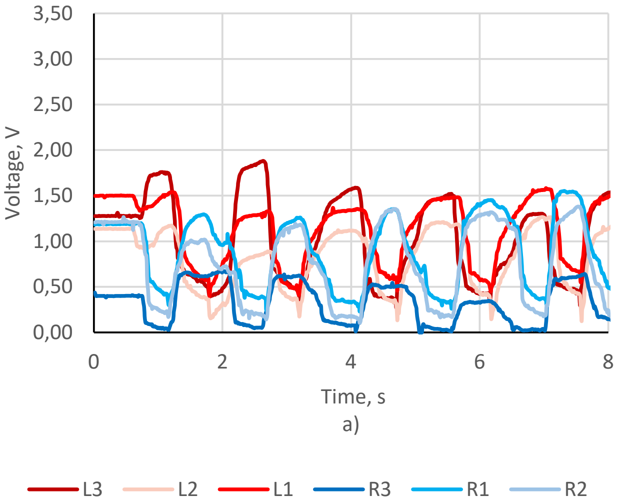

a)

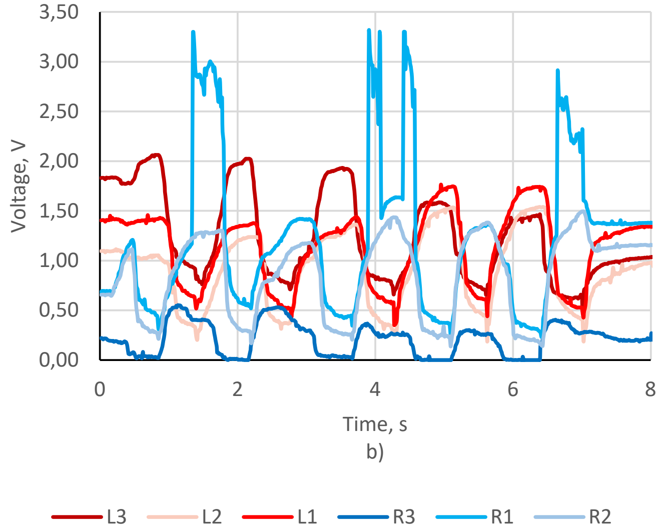

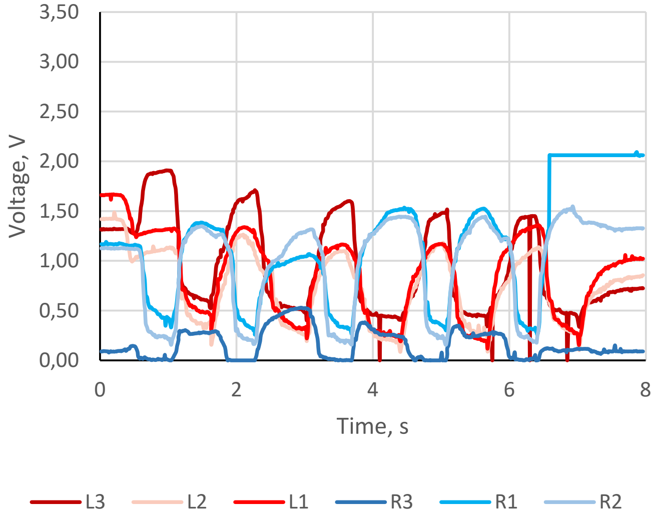

Figure S6: Walk with one straight leg gate phase, first, second and third try
